# Functional testing of thousands of osteoarthritis-associated variants for regulatory activity

**DOI:** 10.1038/s41467-019-10439-y

**Published:** 2019-06-04

**Authors:** Jason C. Klein, Aidan Keith, Sarah J. Rice, Colin Shepherd, Vikram Agarwal, John Loughlin, Jay Shendure

**Affiliations:** 1 Department of Genome Sciences, University of Washington, Seattle, WA 98195 USA; 2 Skeletal Research Group, Institute of Genetic Medicine, Newcastle University, International Centre for Life, Newcastle-upon-Tyne, NE1 3BZ UK; 3 Brotman Baty Institute for Precision Medicine, Seattle, WA 98195 USA; 4 Howard Hughes Medical Institute, University of Washington, Seattle, WA 98195 USA

**Keywords:** Functional genomics, Gene expression, Gene regulation, Osteoarthritis

## Abstract

To date, genome-wide association studies have implicated at least 35 loci in osteoarthritis but, due to linkage disequilibrium, the specific variants underlying these associations and the mechanisms by which they contribute to disease risk have yet to be pinpointed. Here, we functionally test 1,605 single nucleotide variants associated with osteoarthritis for regulatory activity using a massively parallel reporter assay. We identify six single nucleotide polymorphisms (SNPs) with differential regulatory activity between the major and minor alleles. We show that the most significant SNP, rs4730222, exhibits differential nuclear protein binding in electrophoretic mobility shift assays and drives increased expression of an alternative isoform of *HBP1* in a heterozygote chondrosarcoma cell line, in a CRISPR-edited osteosarcoma cell line, and in chondrocytes derived from osteoarthritis patients. This study provides a framework for prioritization of GWAS variants and highlights a role of *HBP1* and Wnt signaling in osteoarthritis pathogenesis.

## Abstract (web summary)

GWAS have identified risk loci for osteoarthritis (OA), but the causal variants still have to be determined. Here, the authors apply a massively-parallel reporter assay to screen 1,605 candidate SNPs in 35 OA loci, which prioritizes six SNPs in four loci, one of which, rs4730222, is characterized in more detail.

## Introduction

Osteoarthritis (OA) is a chronic disease of the articulating joints, characterized by the degradation of articular cartilage, synovial inflammation, and remodeling of the subchondral bone. This leads to a debilitating impairment of joint function. OA is the most common cause of disability in adults over the age of 60, and it is predicted that by 2050, it will affect over 130 million individuals worldwide^1^. Genetic factors comprise ~50% of an individual’s total risk of developing the disease. A series of genome-wide association studies (GWAS) have been carried out in OA and have identified 35 independent genetic signals for disease susceptibility. Outside of OA, GWAS have successfully implicated thousands of genetic loci in a wide range of common human diseases. Most of the underlying signal is believed to derive from variation in non-coding regulatory sequences. However, because of linkage disequilibrium (LD), it has been extraordinarily challenging for the field to identify the variants that causally underlie each association.

Over the past decade, we and others developed massively parallel reporter assays (MPRAs) to increase the throughput at which regulatory sequences can be tested for functional potential^2–5^. An MPRA involves cloning thousands of candidate regulatory sequences to a single reporter gene, transfecting them to a cell line en masse, and performing deep sequencing of the resulting transcripts to quantify the degree of transcriptional activation mediated by each candidate regulatory sequence. MPRAs have previously been applied to characterize variants underlying eQTLs (in LCLs)^6^, red blood cell traits (in K562 and human erythroid progenitors/precursors)^7^, cancer-associated common variants (in HEK293 cells)^8^, and adiposity-associated common variants (in HepG2 cells^9^).

Here, we apply an MPRA (specifically, STARR-seq^5^) to quantify the relative regulatory potential of SNPs residing on haplotypes implicated in (OA), prioritizing six out of 1,605 SNPs with differential regulatory activity. The most significant SNP, rs4730222 falls within an alternative TSS of *HBP1*, shows differential nuclear protein binding, and leads to increased expression of an alternative isoform(s) of *HBP1* in several systems.

## Results

### MPRA to prioritize regulatory GWAS variants

**Fig. 1:**
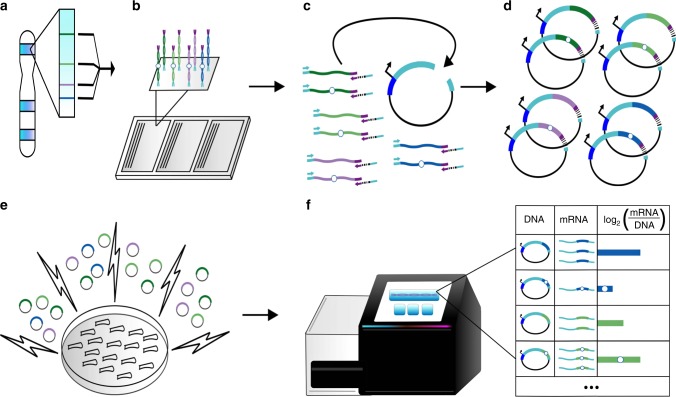
Schematic of massively parallel reporter assay. **a** For each GWAS-lead SNP, we identified all SNPs in LD with r^2^ > 0.8. Colored lines indicate SNPs in the same LD block. **b** For all SNPs, we extracted 196 nt of genomic sequence centered at the SNP, and separately synthesized the minor (hollow circle) and major alleles, flanked by common adaptor sequences (cyan and purple). **c**, **d** We amplified our library from the array via PCR with primers directed at the common adaptors, in the process appending five nt degenerate barcodes (black lines) and additional sequences homologous to the vector (cyan). We cloned our barcoded library of all major and minor alleles into the STARR-seq vector. Each putative regulatory region is cloned into the 3′-UTR of a reporter gene (cyan) with a minimal promoter (dark blue). **e** We transfected our library into Saos-2 cells via electroporation. Forty-eight hours post transfection, we extracted RNA and DNA. **f** We determined the abundance of each allele–barcode combination in the mRNA and DNA population through sequencing. For each allele, we calculated one activity score as the average log_2_(RNA/DNA) across all independent measurements

We compiled a list of 35 lead SNPs associated with OA in Europeans via GWAS, with minor allele frequencies over 5%^10–27^. Each SNP represents an independent signal with *p* < 5e-8 (genome-wide significant; *n* = 20) or *p* < 5e-5 (genome-wide suggestive; *n* = 15) (Supplementary Table 1). We identified all SNPs in LD with an r^2^ > 0.8 in Europeans using rAggr (Fig. 1a), resulting in a list of 1,605 candidate SNPs. For the major and minor allele of each SNP, we synthesized 196 nucleotides (nt) of genomic sequence, centered on the SNP and flanked by adaptor sequences, on a microarray (230 nt oligos; Fig. 1b; Supplementary Data 1).

**Fig. 2:**
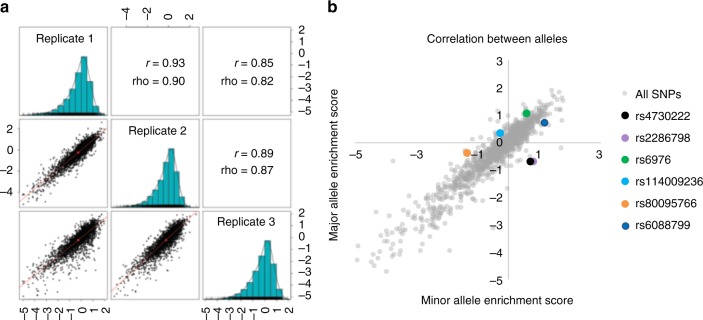
Pairwise correlations between replicates and identification of alleles with differential regulatory activity. **a** Pairwise correlation of RNA/DNA ratios between three replicates. The lower left triangle shows pairwise scatter plots. The diagonal provides replicate names and the respective histogram of the RNA/DNA ratios for that replicate. The upper triangle provides Pearson (rho) and Spearman (r) correlation coefficients. **b** Scatter plot of the major versus minor allele for each SNP. Six SNPs with differential regulatory activity (indicated by colors in the legend) were identified using a Mann–Whitney U Test with an FDR of 10%. Source data are provided as a Source Data file

In order to increase quantitativeness of the assay, we modified STARR-seq by incorporating five nt degenerate barcodes during PCR amplification of array-derived oligonucleotides, i.e., prior to cloning. This modification allowed us to effectively make multiple independent measurements (unique barcode–allele combinations) of each allele in a single experiment (Fig. 1c)^5^. We then cloned these barcoded oligos into the human STARR-seq vector and transfected Saos-2 cells, an osteosarcoma cell line, in triplicate. Importantly, in STARR-seq, the candidate regulatory sequences are located within the transcript itself, rather than upstream of the promoter as in a conventional reporter assay (Fig. 1d). From the transfected Saos-2 cells, we extracted, amplified, and sequenced both DNA and RNA corresponding to the cloned region, and then calculated activity scores as the normalized log_2_ (proportion of RNA reads/proportion of DNA reads) for each barcode–allele combination (Fig. 1e, f). For all alleles with at least three independent barcodes (i.e., a barcode–allele pair in any of the three replicates), we averaged the allele activity scores to a single value. Due to bottlenecking during library construction, some alleles were under sampled and therefore excluded from further analysis. Altogether, we obtained activity scores for 2,537 of 3,210 alleles (79%; avg. 12.9 measurements per allele) (Supplementary Data 2), and activity scores for both alleles of 1,132 of 1,605 SNPs (70%; avg. 13.4 measurements per allele) (Supplementary Data 3). Enrichment scores were highly correlated between independent transfections, with Spearman rho’s ranging from 0.82 to 0.9 and Pearson r’s ranging from 0.85 to 0.93 (Fig. 2a; Supplementary Data 4).

### Enrichment of regulatory marks in MPRA active sequences

We first asked whether these STARR-seq-based activity scores correlated with biochemical marks for putative enhancers. For this analysis, we looked at all major alleles with at least three independent barcodes (*n* = 1,254). We ranked these major alleles into quintiles, and then intersected these with data sets of biochemical marks of putative enhancers in cartilage and bone (H3K27ac in bone marrow-derived chondrocytes, H3K27ac in the human embryonic limb buds, and ATAC-seq in knee OA cartilage)^28,29^ (Supplementary Fig. 1). The highest scoring quintile was significantly enriched for overlap with H3K27ac ChIP-seq peaks in embryonic limb bud from E41 (2.1-fold, Bonferroni-corrected chi-square *p* = 0.035), E44 (2.0-fold, Bonferroni-corrected chi-square *p* = 0.031), and E47 (2.3-fold, Bonferroni-corrected chi-square *p* = 0.0039), relative to all other major alleles. While the trend remained, there was neither a significant enrichment in knee OA cartilage ATAC-seq peaks (1.7-fold) nor bone marrow-derived chondrocyte H3K27ac peaks (1.4-fold). The highest scoring quintile includes 251 genomic regions, 65 of which overlap putative enhancers from at least one data set. In contrast, the lowest scoring quintile includes 250 genomic regions, only 37 of which overlap putative enhancers from at least one data set (1.7-fold enrichment, chi-square *p* = 2.0e-3). Altogether, these enrichments demonstrate that at least a subset of the 1,605 genomic regions tested here correspond to enhancers in OA-relevant tissues.

To evaluate whether these enrichments were specific to bone and cartilage, we similarly tested for overlap with H3K27ac ChIP-seq peaks in H1-hESCs, HeLa, HepG2, Gm12878, K562, and HSMM cell lines. We identified significant enrichment in H1-hESC (1.5-fold, Bonferroni-corrected chi-square *p* = 0.0021), HeLa (1.8-fold, Bonferroni-corrected chi-square *p* = 0.0038), and HepG2 (1.6-fold, Bonferroni-corrected chi-square *p* = 0.023) cells, and non-significant trends in Gm12878 (1.5-fold), K562 (1.3-fold), and Hsmm (1.1-fold). Although weaker in magnitude than the overlap with embryonic limb bud H3K27ac ChIP-seq peaks, these trends suggest that many of the tested genomic regions are not cell-type specific.

To our knowledge, this data set is the largest functional screen for enhancers in an osteosarcoma cell line. Therefore, we asked whether we could use our data to identify TFs that contribute to enhancer activity in bone. Toward the goal of predicting the enhancer activity of the major allele, we trained a tenfold cross-validated lasso regression model that considered 853 evolutionary, biochemical, and sequence-derived features (Supplementary Data 5). The predictions of our model correlated to the observed enhancer activity scores with a Spearman rho of 0.28 (Supplementary Fig. 2A). Training the lasso regression model on the entire data set, 76 total features were selected by the model (Supplementary Data 6, Supplementary Table 2). The 30 top features, which include both histone marks and TF-binding predictions of the corresponding genomic regions, are shown in Supplementary Fig. 2B.

### Differential regulatory activity of SNPs in MPRA

We next sought to ask whether any alleles are differentially active, focusing on the 1,132 SNPs for which we successfully measured activity scores for both alleles (Supplementary Data 2). Overall, activity scores for two alleles of a given SNP were highly correlated, with an overall Spearman correlation of 0.744 (Fig. 2b). This was reassuring, given that each pair of alleles was separately synthesized and cloned, and therefore present at non-identical abundances in the STARR-seq library. After correcting for multiple testing with Benjamini–Hochberg (BH) at a 10% FDR, we identified six SNPs whose alleles demonstrated significantly differential functional activity in Saos-2 cells (Fig. 2b; Supplementary Data 2). The most significant SNP, rs4730222, is located in the 5′-UTR of several non-canonical isoforms of HMG-Box transcription factor 1 (*HBP1*). Other significant SNPs include rs2286798 (intronic to *ITIH1*), rs6976 (3′-UTR of *GLT8D1*), rs114009236 (intronic to *SUPT3H*), rs80095766 (intronic to *COG5*), and rs6088799 (intronic to *UQCC*). Two pairs of the six significant SNPs are at the same loci (the ones near *HBP1* and *COG5*, both at chromosome 7q22.3; and the ones near *ITIH1* and *GLT8D1*, both at chromosome 3p21.1). To further validate these six SNPs, we tested each of the six in a conventional luciferase-based enhancer assay, also in Saos-2 cells. The results for rs4730222 and rs6088799 were concordant with STARR-seq results, while the results for rs80095766 also showed differential activity, but in the opposite direction to STARR-seq. The other three SNPs did not demonstrate significantly differential activity.

Three of the six SNPs (notably the three with the most significant differential expression) are predicted to disrupt protein binding by RegulomeDb, compared with 117/1,126 of the remaining SNPs (*p* = 1.67e-3, chi-square). rs4730222 is predicted to disrupt binding of several proteins based on ChIP-seq, including *POLR2A* (Supplementary Data 7). rs2286798 disrupts an *En1* motif, and serves as an eQTL for *WDR51A, FLJ12442, ITIH3*, and *NT5DC2* in lymphoblastoid cells and *FLJ12442* and *ITIH4* in monocytes (Supplementary Table 3). rs6976 is predicted to disrupt binding of several proteins based on ChIP-seq, including *POLR2A* and *EP300* (Supplementary Table 4). It is also an eQTL for *WDR51A, FLJ12442*, and *NT5DC2* in lymphoblastoid cells and *FLJ12442* and *ITIH4* in monocytes.

### Validation and characterization of rs4730222

We chose to further characterize rs4730222 for several reasons. First, this SNP was the most significant hit from our reporter assay, with a substantial difference in activity between the two alleles (2.5-fold increased expression of the minor, disease-associated allele; BH-adjusted *p*-value = 8.8e-5). Second, its differential activity was confirmed by a conventional luciferase-based enhancer assay (2.02-fold, Wilcoxon matched-pairs signed rank test *p* < 1e-4). Third, of the six, only rs4730222 overlaps with putative enhancer marks in embryonic limb buds (H3K27ac) and osteoarthritic knee cartilage (ATAC-seq). Fourth, we had previously prioritized *HBP1* as a potential target in the 7q22 osteoarthritis-associated locus based on gene expression analysis^30^. HBP1 is a transcriptional repressor that regulates the Wnt-beta–catenin pathway as well as superoxide production, both of which have been implicated in OA development and progression^31–33^.

**Fig. 3:**
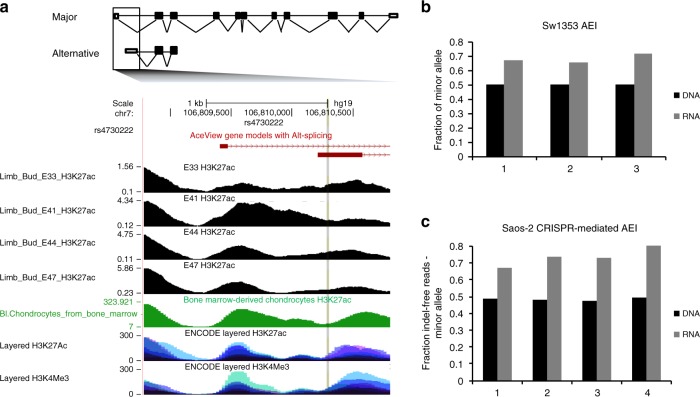
Functional validation of rs4730222. **a** Gene model of *HBP1* ensemble isoform ENST00000468410 (major) and ENST00000497535 (alternative). UCSC genome browser zoomed in to the two transcriptional start sites^57^. Rs4730222, within the 5′-UTR of the alternative transcript, is indicated by a vertical gray line traversing the annotation tracks. The four black tracks are H3K27ac performed in human embryonic limb bud at E33, E41, E44, and E47, respectively^28^. The green track is H3K27ac data from chondrocytes derived from cultured bone marrow mesenchymal stem cells^29^. Layered H3K27ac is H3K27ac ChIP-seq (a marker for active enhancers and active promoters) layered from GM12878, H1-hESC, HSMM, HUVEC, K562, NHEK, and NHLF cells. Layered H3K4me3 is H3K4me3 ChIP-seq (marker for active promoters) layered from the same seven ENCODE cell lines. **b** Allelic expression imbalance in SW1353, a chondrosarcoma cell line heterozygote for rs4730222. Black bars represent the fraction of the minor allele in DNA, and gray bars indicate the fraction of the minor allele in cDNA. *n* = 3 independent experiments. **c** Allelic expression imbalance in Saos-2 cells with the minor allele of rs4730222 introduced through CRISPR-mediated HDR. Bars are the same as in Fig. 3B. *n* = 4 independent experiments. Source data are provided as a Source Data file

rs4730222 overlaps with several marks associated with active regulatory DNA: H3K27ac (mark for active enhancers and promoters) from bone marrow-derived chondrocytes^29^, human embryonic limb bud at E33, E41, E44, and E47^28^, and ENCODE layered data; H3K4me3 (mark for active promoters) in ENCODE layered data; and ATAC-seq (mark for open chromatin) peaks from articular knee cartilage of OA patients^34^ (Fig. 3a). *HBP1* contains several transcript isoforms, with three probable alternative promoters identified from cap-selected clones and nine validated alternative polyadenylation sites^35^. One of the three probable alternative promoters contains rs4730222 at position + 80 relative to the alternative transcriptional start site (TSS). We therefore hypothesized that the variant may alter expression of this or another *HBP1* isoform. To test this, we first confirmed that the alternative TSS overlapping rs4730222 is utilized in Saos-2 and SW1353 cells (a chondrosarcoma cell line) with qRT-PCR with primers contained within this 5′-UTR as well as spanning to the following exon. However, despite multiple attempts, we failed to successfully amplify the isoform from the alternative TSS to the canonical stop, suggesting that the alternative TSS may belong to a truncated isoform of the *HBP1* transcript. Based on PCR and Sanger sequencing, the truncated isoform is most likely ENST00000497535, which contains the alternative 5′-UTR and first two exons (Fig. 3a; Supplementary Fig. 3).

After confirming that rs4730222 is transcribed as part of a *HBP1* isoform in osteogenic and chondrogenic cells (albeit not to the canonical stop), we genotyped rs4730222 in several cell lines (SW1353, Tc28a/2, Saos-2, chondrogenic progenitor cells) in hopes of identifying a heterozygous line. As SW1353 was heterozygous for rs4730222, we tested it for allelic expression imbalance (AEI) of the transcribed SNP. In all three biological replicates, we observed a significant allelic imbalance (Fisher’s exact test, *p* < 1e-5), with the minor allele showing a 1.3 to 1.4-fold relative enrichment in RNA/DNA compared with the major allele. This was more modest but directionally concordant with 2.5-fold enrichment of the minor allele in the reporter assay (Fig. 3b).

The OA-implicated haplotype at 7q22.3 is a ~500 -kb region of high-linkage disequilibrium, and consequently accounts for 283 of the 1,605 SNPs tested here. The observed AEI of the *HBP1* alternative isoform in SW1353 cells, which are likely heterozygous for the entire OA-associated haplotype, could be consequent to any or several of these SNPs, or to other forms of genetic variation. To test whether rs4730222 causally underlies the allelic imbalance of the *HBP1* alternative transcript, we introduced the minor allele of rs4730222 into Saos-2 cells, which are homozygous for the major allele, through CRISPR-mediated homology directed repair (HDR). We generated four biological replicates (i.e., independently edited cell populations), and quantified the RNA/DNA ratio of indel-free reads derived from the major versus minor allele. Similar to the SW1353 AEI (1.3 to 1.4-fold), we identified a 1.4 to 1.6-fold relative enrichment in the RNA/DNA ratio for the minor allele compared with the major allele (Fisher’s exact test, *p* < 1e-5; Fig. 3c). This confirms that the minor allele at rs4730222 causally underlies upregulation of the *HBP1* alternative isoform.

We next sought to test whether rs4730222 is associated with different levels of the *HBP1* alternative isoform in osteoarthritic tissue. To do so, we tested for AEI in chondrocytes derived from OA patients. For each of nine patients heterozygous for rs4730222, we extracted RNA and DNA and amplified and sequenced the 5′-UTR containing the SNP (three technical replicates per patient) from DNA and cDNA. Despite low cDNA concentrations and low expression of the alternative TSS, we observed an overall trend in AEI in accordance with our reporter assay and cell models (median AEI = 1.06, Mann–Whitney U-test on 9 DNA ratios vs. 9 RNA ratios, *p* = 0.008). Interestingly, one patient appeared to exhibit AEI in the opposite direction^36,37^ (Supplementary Fig. 4).

Finally, in order to test whether rs4730222 is driving differential expression through TF binding, we performed EMSA experiments using nuclear protein from both Saos-2 and SW1353. For both cell types, we identified reproducible differential binding of multiple protein complexes, with greater binding for the major, G allele (Supplementary Fig. 5).

Speculating that its effect might be on the promoter of the alternative isoform of *HBP1*, we subjected the same fragment containing rs4730222 from the luciferase-based enhancer assay to a conventional luciferase-based promoter assay. We once again observed significant, differential expression of the two alleles, but to our surprise the directionality of effect was opposite to that of the luciferase-based enhancer assay, the STARR-seq experiment, as well as all of the other aforementioned experiments, suggesting that in this context, the regulatory element containing the SNP acts as an enhancer rather than promoter (Supplementary Fig. 6).

In sum, multiple independent lines of directionally consistent evidence, including the STARR-seq MPRA, predicted protein binding, observations of AEI in both a chondrosarcoma cell line and OA patient chondrocytes, as well as with CRISPR/Cas9-mediated introduction of the exact allele into an osteosarcoma cell line, and finally direct demonstration of differential protein binding, all support the case for rs4730222 as having differential regulatory activity between the major and minor alleles, likely by impacting expression of an alternative isoform of *HBP1*. Of note, testing rs4730222 in enhancer versus promoter luciferase-based assays yielded directionally discordant results, highlighting the importance of context in reporter assays.

## Discussion

In summary, we set out to functionally test the regulatory effects of 1,605 SNPs that potentially underlie 35 GWAS signals for osteoarthritis. We succeeded in generating reproducible measurements of differential regulatory activity for almost three quarters of the regions tested. While this data set captures most SNPs around our GWAS candidates, due to technological limitations, it is possible that potentially causal variants in these regions were not included in our assay. The most highly active regions in our assay were ~2-fold enriched for biochemical marks associated with enhancers. Considering over 800 features, we built a predictive model of enhancer activity in Saos-2, whose predictions correlated with the observed enhancer activity scores with a Spearman rho of 0.28. Some of the top features include FLI1 and p53, which have been associated with Ewing’s sarcoma and osteosarcoma, respectively. NR2F1, a transcriptional repressor that is overexpressed in bone^38^, was negatively correlated with enhancer activity (Supplementary Fig. 2B). Surprisingly, the top predictor was H3K27me3, which is conventionally associated with heterochromatin. One possible explanation is that the Polycomb repressive complex 2 (PRC2), which mediates the introduction of this mark, is frequently dysregulated in osteosarcoma cell lines^39^. An alternative explanation is that these elements are being tested outside of their native chromosomal context, and may act as poised enhancers in the genome. A further caveat is that the cell types in which the ChIP-seq data were generated (e.g., osteoblasts and EWS502 for H3K27me3) are not identical to the cell line in which the MPRA data were generated (i.e., Saos-2).

We identified six SNPs that were reproducibly associated with differential expression in the STARR-seq assay at an FDR of 10%, three of which are predicted to alter protein binding. The most significant of these, rs4730222, resides in the 5′-UTR of multiple isoforms of *HBP1*, a transcriptional repressor. The minor, OA-associated risk allele of rs4730222 appears to functionally underlie increased transcriptional levels of alternative isoform(s) of *HBP1*. This conclusion is supported by multiple lines of evidence, including the STARR-seq MPRA, observations of AEI in both a chondrosarcoma cell line and OA patient chondrocytes, and CRISPR/Cas9-mediated introduction of the exact allele into an osteosarcoma cell line. Notably, testing a fragment containing rs4730222 in a enhancer luciferase-based assay yielded a result that was directionally concordant with all other experiments, while testing the same segment in a promoter assay yielded a directionally opposite effect. In our view, the directionality observed with the CRISPR/Cas9-mediated introduction of the exact allele takes primacy, as it reflects the native chromosomal context and furthermore isolates the SNP in question from other SNPs in linkage disequilibrium.

We further demonstrated that the SNP at rs4730222 results in differential protein binding in both osteosarcoma and chondrosarcoma cells, suggesting a mechanism for the differential expression. As we note more binding at the major G allele, which results in decreased expression of the alternative *HBP1* transcript, we propose that the C allele is displacing a transcriptional repressor. There are several potential transcriptional repressors bound at rs4730222 in different cell lines based on ChIP-seq data, including ZBTB7A, YY1, MAX, GTSAP30, NFKB1, and ZBTB33. Of note, ZBTB33 has previously been noted to repress non-canonical Wnt signaling^40^. The full list of ChIP-seq peaks at rs4730222 identified from RegulomeDb is provided as Supplementary Data 7.

We performed our assay in Saos-2 cells, an osteosarcoma cell line. Therefore, our enrichment scores are a function of this particular *trans* environment. Although OA is characterized by the loss of articular cartilage, there are concurrent pathological changes to other joint tissues, including bone, making an osteosarcoma cell line relevant to OA pathogenesis^41^. These take the form of subchondral sclerosis and the generation of osteophytes. Furthermore, there is growing evidence that aberrant development of the joint, including in the transition from articular chondrocyte to hypertrophic chondrocyte and the subsequent ossification of the cartilage, also predisposes to OA^42^. Moreover, we validated the AEI and differential protein binding of our lead SNP, rs4730222 in both Saos-2 and SW1353, a chondrosarcoma cell line, suggesting that this particular finding is relevant to both bone and cartilage-derived cells.

We propose that rs4730222 is contributing to osteoarthritis by disrupting the tightly regulated Wnt signaling pathway. Wnt signaling plays a key role in joint development^43^, as well as in joint destruction in the form of osteoarthritis^44^. The Wnt pathway is tightly regulated in both bone and cartilage, in order to allow for proper joint development and homeostasis^45^. Too little Wnt signaling disrupts both cartilage maintenance and subchondral bone homeostasis, and contributes to OA in animal models^45,46^. Similarly, excess signaling also triggers disease, and leads to an OA phenotype in mice^47^. HBP1 acts as a negative regulator of the Wnt-beta–catenin pathway^48^, and therefore its altered expression secondary to rs4730222 may disrupt the tight regulation of Wnt signaling and predispose to OA.

While we demonstrate a functional role for rs4730222 on HBP1 expression, further experiments are needed to directly implicate rs4730222 in Wnt signaling and OA pathogenesis. In this work, we edited the rs4730222 allele in Saos-2 in a bulk population and differentiated the editing outcomes through sequencing. Looking forward, it will be necessary to generate monoclonally edited cell lines, e.g., in Saos-2 and SW1353, to perform further functional investigation of the role of this variant. For example, the functional consequences of the variant could be examined through transcriptome sequencing and pathway analysis, or its potential impact in Wnt signaling and/or OA pathogenesis analyzed through the TCF/LEF reporter assay or qPCR of marker genes.

We previously described reduced expression of the canonical *HBP1* transcript in OA tissue, relative to healthy tissue^30^. However, the increased expression was not consistently linked to a specific allele, suggesting that it may be driven by a *trans-*activating factor, rather than a *cis-*regulatory element. Furthermore, our findings here pertain to an alternative isoform of *HBP1*, not the canonical transcript. Indeed, we do not observe AEI of the canonical *HBP1* transcript, but rather only of the alternative isoform.

We speculate that the impact of rs4730222, wherein the disease-associated allele increases expression of an alternative transcript, secondarily reduces expression of canonical *HBP1*. One possibility is that the alternative isoform encodes a non-coding RNA with an upstream open-reading frame (uORF). This isoform of *HBP1* may have a *trans* effect on the canonical transcript, or have its own, yet uncharacterized, function in the cell. In addition, alternative TSSs have been shown to modulate translational efficiency and tissue specificity of genes^49,50^. Through one or several of these mechanisms, the expression levels of this isoform might modulate HBP1 levels in certain tissues. Finally, we note that the rs4730222-overlapping isoform expressed in SW1353 and Saos-2 is a truncated version of *HBP1*. Therefore, it may disrupt endogenous activity of the protein, e.g. by acting as a dominant negative. Distinguishing between these mechanistic possibilities for rs4730222 and *HBP1*, as well as for other MPRA-prioritized OA-associated SNPs, should be a high priority for the field.

## Methods

### Identification and design of target SNPs

We selected SNPs that had a minor allele frequency >5% and had been reported as being associated with OA in European populations at a significance level that surpassed or approached the genome-wide threshold of <5e-8. The deadline date for inclusion was May 2017. In total, 35 SNPs were identified, each representing an independent association signal (Supplementary Table 1). We ran rAGGr on our list of 35 candidate SNPs to identify all variants with a minimum minor allele frequency > = 0.001 in linkage disequilibrium with an r^2^ > 0.8 in Europeans (CEU + FIN + GBR + IBS + TSI) based on 1000 Genomes, Phase 3, Oct 2014. We then filtered out any polymorphisms greater than one nucleotide, resulting in a list of 1,605 SNPs. For each variant, we extracted 196 nt of genomic sequence centered around the SNP using BEDTOOLS getfasta, and edited the SNP to create both the minor and major alleles (3,210 sequences). To each 196 nt sequence, we appended HSS_clon_F (5′-TCTAGAGCATGCACCGG-3′) to the 5′ end and DO_R6 (5′-GCCGGTCAGAATGATGG-3′) to the 3′ end. We then ordered the 3,210 sequences in duplicate as part of an Agilent 244 K 230-mer array.

### Library generation

We amplified our sequences off of the Agilent array with HSS_clon_F and R6_5N_HSSR (5′-CCGGCCGAATTCGTCGANNNNNCCATCATTCTGACCGGC-3′) using KAPA HiFi HotStart ReadyMix in a 50 µL reaction with 0.75 ng of DNA and SYBR Green on a MiniOpticon Real-Time PCR system (Bio-Rad) and stopped the reaction before plateauing (13 cycles). This reaction amplified our library, added a five nt degenerate barcode to each sequence, and added both adapters for cloning into the human STARR-seq vector. We purified the PCR product using a 1.5x AMPure cleanup following manufacturer’s protocol. We then ligated 6 ng of our purified PCR into 25 ng of linearized human STARR-seq backbone using the NEBuilder HiFi DNA Assembly Cloning Kit following the manufacturer’s protocol. We transformed 1.2 µL of the ligation product in 50 µL of NEB C3020 cells, grew up overnight in 100 mL of LB + Amp, and extracted the library using a Zymo Research ZymoPURE Plasmid Midiprep Kit.

### STARR-seq screen

We transfected 1.5 million Saos-2 cells (ATCC) with 20 µg of our library in triplicate using the Thermo Fisher Scientific Neon Transfection System with resuspension buffer R at 1250 V, 40 ms, 1 shock, with 100 µL pipettes, in triplicate. After electroporation, we added the cells to 10 -cm plates with pre-warmed media (McCoy’s 5 A with 10% FBS and 1x Pen/Strep). Forty-eight hours post transfection, we extracted both DNA and RNA from each replicate using the Qiagen ALLPrep DNA/RNA Mini Kit. DNA was eluted in 80 µL and RNA was eluted in 30 µL. RNA was treated with Thermo Fisher Scientific TURBO DNase following the manufacturer’s protocol and reverse transcribed using Thermo Fisher Scientific SuperScript III Reverse Transcriptase in a 20 µL reaction with 8 µL of RNA. For each replicate, we amplified DNA in two reactions, each with 2 µg of DNA using NEBNext High Fidelity 2X PCR Master Mix with primers HSS_NF_pu1 (5′-CTAAATGGCTGTGAGAGAGCTCAGGTACAACTGATCTAGAGCATGCACC-3′) and HSS_R_pu1 (5′-ACTTTATCAATCTCGCTCCAAACCCTTATCATGTCTGCTCGAAGC-3′), and stopped before plateauing (15 cycles). After PCR, products were purified with a 1.5x AMPure cleanup, and pooled together. For each replicate, we also amplified cDNA in two reactions, each with 10 µL of RT product in 50 µL reactions with NEBNext High Fidelity 2X PCR Master Mix with primers HSS_F_pu1 (5′- CTAAATGGCTGTGAGAGAGCTCAGGGGCCAGCTGTTGGGGTGTCCAC-3′) and HSS_R_pu1 (5′- ACTTTATCAATCTCGCTCCAAACCCTTATCATGTCTGCTCGAAGC-3′), and stopped before plateauing (18–20 cycles). After PCR, products were purified with a 1.5x AMPure cleanup, eluted in 50 µL each, and pooled together. For the cDNA samples, we performed a nested reaction using KAPA HiFi HotStart ReadyMix in a 50 µL reaction with 1 µL of the pooled outer PCR reaction with HSS-NF-pu1 and pu1R (5′-ACTTTATCAATCTCGCTCCAAACC-3′), and stopped before plateauing (7 cycles). Reactions were purified with a 1.5x AMPure cleanup and eluted in 50 µL each. Flow cell adapters and indexes were added to all DNA and cDNA reactions through an additional round of PCR using Kapa HiFi HotStart ReadyMix in 50 µL reactions with 1 µL of the first DNA PCR or 1 µL of the inner cDNA PCR using an indexed pu1_P5 primer (5′-AATGATACGGCGACCACCGAGATCTACACNNNNNNNNNNACGTAGGCCTAAATGGCTGTGAGAGAGCTCAG-3′) and an indexed pu1_P7 primer (5′-CAAGCAGAAGACGGCATACGAGATNNNNNNNNNGACCGTCGGCACTTTATCAATCTCGCTCCAAACC-3′), and stopped before plateauing (six cycles). The libraries were sequenced on an Illumina NextSeq 500/550 v2 300 cycle mid-output kit.

### Analysis of STARR-seq screen

We aligned all sequencing reads to a reference fasta file of our variants using BWA mem and extracted reads from error-free molecules^51^. Each variant contained several different 5 nt barcodes added through PCR. We counted the number of reads from each replicate for each variant–barcode combination in the DNA and cDNA pool. If there were at least ten DNA reads and at least one RNA read, we calculated an activity score as the log_2_ (number of RNA reads from the variant–barcode combination normalized to the total number of RNA reads, divided by the number of DNA reads from the variant–barcode combination normalized to the total number of DNA reads). We then combined all variant–barcode activity scores from each replicate, and for any variant with at least three different measurements, we averaged the activity score for a final activity score for each variant. This resulted in activity scores for 2,537 of the 3,210 alleles. In total, 1,132 of the 1,605 variants had activity scores for both alleles. For each of the 1,132 variants with activity scores for both alleles, we tested whether the two alleles drove different expression by performing a Mann–Whitney U Test for each variant using SciPy v0.19.1 with Python v2.7.3. We then performed a BH correction with an FDR = 0.10 to correct for multiple testing.

### Luciferase assays

To generate the constructs for use in the luciferase reporter gene assay, primers including either a *KpnI*, *HindIII*, or *XhoI* 5′ restriction were designed to amplify DNA regions encompassing each of the six SNPs. Primer sequences are listed in Supplementary Table 7. Template DNA consisted of a mix of blood DNA from 20 patients. PCR products were TOPO cloned and positive clones were selected using blue/white screening and genotyped by Sanger sequencing to identify clones containing each of the two alleles at the six SNPs. It was confirmed that no other polymorphisms were present in the cloned regions. The required inserts were then sub-cloned using restriction enzymes into either the pGL3-basic or pGL3-promoter luciferase reporter plasmid (Promega), depending upon the predicted regulatory function of the region determined from the ROADMAP Epigenome database. Positive clones were sequenced to ensure the correct sequence of the constructs.

The Saos-2 human osteosarcoma cell line (ATCC) was transfected for the luciferase assays. Twenty-four hours before transfection, Saos-2 cells were seeded at a density of 8,000 per well in a 96-well plate. Cells were transfected with 100 ng of pGL3 construct DNA and 1.5 ng of Renilla luciferase reporter vector pRL-TK (Promega), using FuGene HD transfection reagent (Promega). After 24 h, the cells were lysed, and luciferase activity was determined using a Dual-Luciferase Reporter Assay System (Promega). Luminescence was measured using a GloMax-Multi Detection System (Promega). Firefly luciferase activity was normalized to the activity of Renilla luciferase. Six technical and 5–6 biologic repeats were performed per construct. *P*-values were calculated using a Wilcoxon matched-pairs signed rank test.

### Allelic imbalance of rs4730222 in SW1353 cells

We first genotyped several osteogenic and chondrogenic cell lines for rs4730222 (SW1353, Tc28a/2, Saos-2, chondrogenic progenitor cells) using rs4730222_sangerF (5′-TACGCAGTTCGAATGAATGGGCTC-3′) and rs4730222_sangerR (5′-AGCTACAAAAACCTGGCTGTCCAC-3′). PCR products were purified with a 1.5x AMPure cleanup and Sanger sequenced with rs4730222_sangerF.

We then tested for allelic imbalance of rs4730222 in the isoforms expressing the SNP in SW1353 (ATCC). We performed three independent DNA and RNA extractions using the Qiagen ALLPrep DNA/RNA Mini Kit. DNA was eluted in 80 µL, and RNA was eluted in 30 µL. RNA was treated with TURBO DNase and reverse transcribed with SuperScript III Reverse Transcriptase. We then amplified the 5′-UTR around rs4730222 from each DNA and cDNA sample using KAPA HiFi HotStart ReadyMix in a 50 µL reaction with 100 ng of DNA or 5 µL of cDNA with HBP1_5UTR_F_pu1 (5′-CTAAATGGCTGTGAGAGAGCTCAGAGTCCGGGCTGCGGTCACATGATG-3′) and HBP1_5UTR_R_pu1 (5′-ACTTTATCAATCTCGCTCCAAACCAGCTACAAAAACCTGGCTGTCCAC-3′), and stopped DNA reactions at 25 cycles and cDNA reactions at 32 cycles. Products were purified using a 1.5x AMPure cleanup, and flow-cell adapters and indexes were added using an indexed pu1_P5 primer and an indexed pu1_P7 primer. Libraries were spiked into a Miseq v2 300 cycle run. Reads were aligned to a fasta reference file using BWA mem, and the number of perfect reads coming from both alleles was quantified from both DNA and cDNA from each replicate.

### CRISPR knock-in of rs4730222 in Saos-2 cells

rs4730222 falls within a potential Cas9 PAM site (5′-ACGCGATGAATGGCGAAAGAG**G**G-3′). We therefore designed a guideRNA that would target rs4730222, so that the minor-allele donor would not be re-cut. We ordered the following oligos from IDT: rs4730222_guideF (5′-CACCGACGCGATGAATGGCGAAAGA-3′) and rs4730222_guideR (5′-AAACTCTTTCGCCATTCATCGCGTC-3′) and followed the Zhang lab protocol to clone them into the px458 plasmid (SpCas9–2A-EGFP and single-guide RNA)^52^.

We created our donor vector in two steps. First, we amplified a 1,459 -bp region around rs4730222 with the following primers, which also append 16 -bp homologous sequence to puc19 onto each side of the amplicon: HBP1_puc19F (5′-TCGGTACCCGGGGATCAAGTAGGAAAGTTTCGGTTGAGGAG-3′) and HBP1_puc19R (5′-TCGACTCTAGAGGATCAACTGAACAGATGACCGACTCTACC-3). We then cloned this into a linearized puc19 plasmid using Clontech’s In-Fusion HD Cloning Kit following the manufacturer’s protocol, transformed into Stellar Competent cells, grew up a single colony and extracted plasmid using the Zymo Research ZymoPURE Plasmid Midiprep Kit. We then re-linearized the puc19-HBP1 wild-type plasmid via PCR with puc19_HBP1-linF (5′-GTGGGGGATGGACTTGGCGTG-3′) and puc19-HBP1-linR (5′-CTCCTCAACCGAAACTTTCCTACTT-3′). We also amplified a small region around rs4730222, while mutating the SNP, using mut_insF (5′-AAGTAGGAAAGTTTCGGTTGAGGAG-3′) and mut_insR (5′-CCAAGTCCATCCCCCACGCTCTTTCGCCATTCATCGCG-3′). We then cloned the mutated insert into puc19-HBP1-wt using the In-Fusion HD Cloning Kit and grew up a single colony with the minor allele at rs4730222 flanked by 600–850 bp of homology on each side.

We transfected 1 million Saos-2 cells with 10 µg of our px458-rs4730222 guide and 10 µg of our donor library containing the minor allele using the Neon Transfection system as described above. Seventy-two hours post transfection, we performed FACS on a BD FACS Aria III to isolate ~150,000 GFP + cells (transfected with px458), which we then expanded. On day 10 post transfection, we extracted DNA and RNA, performed reverse transcription with Superscript III, and amplified the region surrounding rs4730222 from both DNA (using HBP1_5UTR_F_pu1 and HBP_DNA_Routside (5′-TAGGTGGGCAATCCTGGGAGAAGGTAC-3′)), and RNA (using HBP1_5UTR_F_pu1 and HBP_RNA_Routside (5′-TGCCAGATTCTGACTCACTATTTGC-3′)) in 50 µL reactions using KAPA HiFi 2x ReadyMix. We then purified the PCR reactions with a 1.5x AMPure cleanup, eluted in 50 uL, and used 1 µL in a nested reaction with pu1L (5′-CTAAATGGCTGTGAGAGAGCTCAG-3′) and HBP1_5UTR_R_pu1. Reactions were purified with a 1.5x AMPure cleanup, and flow-cell adapters and indexes were added using an indexed pu1_P5 primer and an indexed pu1_P7 primer. Libraries were spiked into a Miseq v2 300 cycle run. Reads were aligned to a fasta reference file using BWA mem, and the number of perfect reads coming from both alleles was quantified from both DNA and cDNA from each replicate.

### Allelic imbalance of rs4730222 in OA patients’ chondrocytes

Cartilage tissue samples were obtained from OA patients who had undergone joint replacement surgery at the Newcastle upon Tyne NHS Foundation Trust hospitals. The Newcastle and North Tyneside Research Ethics Committee granted ethical approval for the collection, with each donor providing verbal and written informed consent (REC reference number 14/NE/1212). Our patient ascertainment criterion has been described in detail previously^53,54^. The cartilage was removed from the joint using a scalpel and was collected distal to the OA lesion. The tissue samples were stored frozen at −80 °C and ground to a powder using a Retsch Mixermill 200 (Retsch Limited) under liquid nitrogen. Nucleic acids were then extracted from the ground tissue using TRIzol reagent (Life Technologies) according to the manufacturer’s instructions, with the upper aqueous phase separated for RNA isolation, while the interphase and lower organic phase were used to isolate DNA. RNA was reverse transcribed using the SuperScript First-Strand cDNA synthesis kit (Invitrogen). Matched DNA and cDNA were amplified with KAPA HiFi 2x ReadyMix and SYBR Green and halted before plateauing. The primers sequences were as follows:

5′-CTAAATGGCTGTGAGAGAGCTCAGAGTCCGGGCTGCGGTCACATGATG-3′ and 5′-ACTTTATCAATCTCGCTCCAAACCAGCTACAAAAACCTGGCTGTCCAC-3′.

All samples were purified with a 1.5x AMPure cleanup following the manufacturer’s instructions and eluted in 50 µL Qiagen Elution Buffer. One microliter of purified product was then indexed for Illumina sequencing using an indexed pu1_P5 primer and an indexed pu1_P7 primer. Libraries were spiked into a Miseq v2 300 cycle run. Following our previously devised processing pipeline^55^, reads were aligned to a fasta reference file using BWA mem^51^, and the number of aligning reads coming from both alleles was quantified from both DNA and cDNA from each replicate. For statistical analysis, a Mann–Whitney U test was performed comparing DNA versus RNA abundances of the minor allele for the 18 values (9 for DNA and 9 for RNA; averaged over three replicates for each of nine patients).

### Electrophoretic mobility shift assays (EMSAs) of rs4730222

Nuclear protein was extracted from SW1353 and Saos-2 cells as previously described^56^. For each allele of rs4730222, forward and reverse single-stranded DY682-labeled oligonucleotides (Eurofins MWG Operon), spanning 15 nt each side of the SNP (Supplementary Table 4), were annealed to generate double stranded probes. EMSAs were performed as previously described^56^.

### Characterization of HBP1 rs4730222-containing isoforms

We designed the following set of primers to differentiate between different HBP1 isoforms: major (1stExonF: 5′-GTGTGGGAAGTGAAGACAAATCAGATGC-3′ and LastExonR: 5′-CTTCCACCTGTCACCAAGGATCACAC-3′), 5′-UTR (UTR_qPCR_F: 5′-CAGTCTCCGCCTTTCAACCTATG-3′ and UTR_qPCR_R: 5′-ATGAACTCGAGTGTAGAGTGCACAG-3′), Truncated (UTR_qPCR_F and Exon6_R: CCACCTCATTTTCACGGTAAGTAG-3′) and Full Len (UTR_qPCR_F and LastExonR). We performed technical triplicates for each qPCR using KAPA Robust 2x Hotstart Readymix with cDNA from wild-type SW1353 cells, letting the reaction go for 40 cycles. We then ran products on a gel, and differentiated between ENST00000497535 and ENST00000485846 based both on size and Sanger sequencing.

### Lasso regression model to predict enhancer activity

For each candidate enhancer, we computed a total of 853 features derived from either (i) the sequence itself, or (ii) experimentally measured information, computed as a mean signal extracted from the corresponding region of the human genome (Supplementary Data 5, 6). The sequence-based features represent the conservation of the sequence, general G/C content, predicted chromatin state, and likelihood of binding to an assortment of transcription factors. In contrast, the experimentally derived features represent empirical measurements of chromatin/epigenetic state and binding to transcription factors. The features were derived from custom Perl scripts, the UCSC genome browser^57^, DeepSEA^58^, DeepBind^59^, and ChIP-Atlas^60^.

Right-skewed data such as ChIP-seq and CAGE signal were log-transformed to approximate a normal distribution, and each feature was then z-score normalized to scale the features similarly. This enabled a direct comparison of coefficients among features derived from the resulting linear models.

We trained a lasso regression model on each of 10-fold of the data^55^. A lasso regression model was chosen specifically because it employs an L1 regularization penalty, which leads to the selection of the fewest features that maximally explain the data. The strength of the regularization was controlled by a single *λ* parameter, which was optimized using 10-fold cross-validation on the entire data set. To evaluate the most relevant features selected, we trained a lasso regression model on the full data set and visualize the top 30 coefficients with the greatest magnitude (Supplementary Fig. 2B). A full table of the selected features and their coefficients are provided (Supplementary Data 6).

## Supplementary information

Supplementary Information

Description of Additional Supplementary Files

Supplementary Data File 1

Supplementary Data File 2

Supplementary Data File 3

Supplementary Data File 4

Supplementary Data File 5

Supplementary Data File 6

Supplementary Data File 7

Source Data

## Data Availability

All processed data are provided in supplementary data files. Raw data are available as SRA accession PRJNA532491. The source data underlying Figs. 2a, 2b, 3b, and 3c and Supplementary Figs 1, 2A, 3A, 4, 5, and 6 are provided as a Source Data file.
