# Supplementary material for: Functional testing of thousands of osteoarthritis-associated variants for regulatory activity
Source: Nat Commun. 2019 Jun 4;10:2434. doi: 10.1038/s41467-019-10439-y (PMC6547687; doi:10.1038/s41467-019-10439-y)
Supplement: Supplementary file 1 — Supplementary Information [file 41467_2019_10439_MOESM1_ESM.pdf]

Functional Testing of Thousands of Osteoarthritis-Associated Variants for Regulatory Activity

Klein et al.

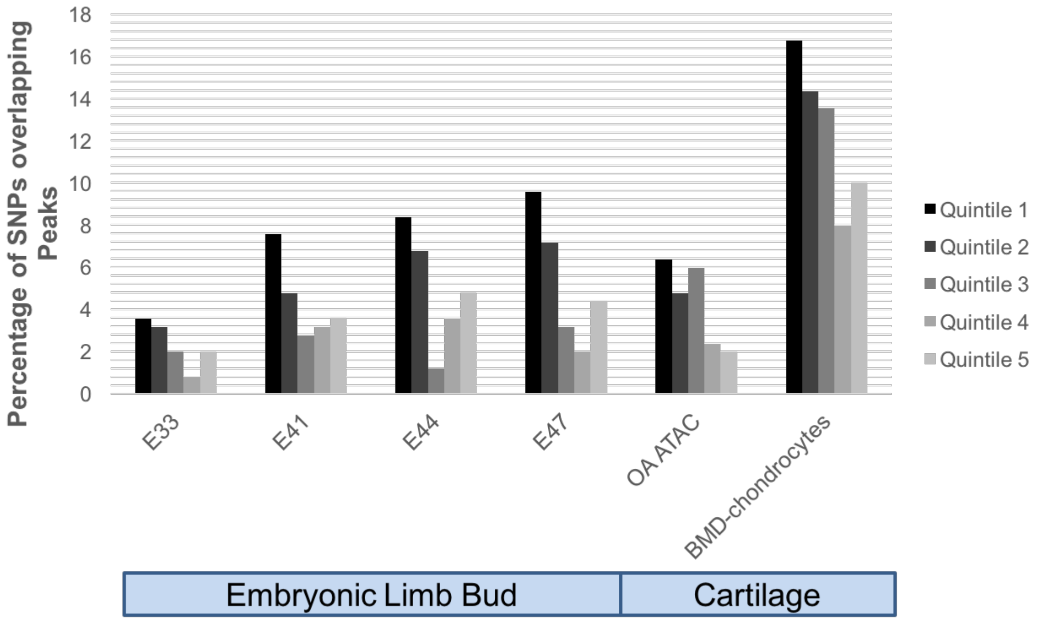

Supplementary Figure 1. Overlap between tested sequences and enhancer marks. The 1,203 SNPs were split into 5 quintiles of 251 or 250 sequences each, based on their normalized RNA/DNA activity score. Quintile 1 refers to the sequences with the highest activity scores and quintile 5 refers to the sequences with the lowest activity scores. We then overlapped each quintile with peaks called from OA ATAC-seq[34], BMD-chondrocytes H3K27ac ChIP-seq[29], and human embryonic limb bud H3K27ac ChIP-seq from E33, E41, E44, and E47[28]. Y-axis is the fraction of SNPs in each quintile overlapping peaks. Source data are provided as a Source Data file.

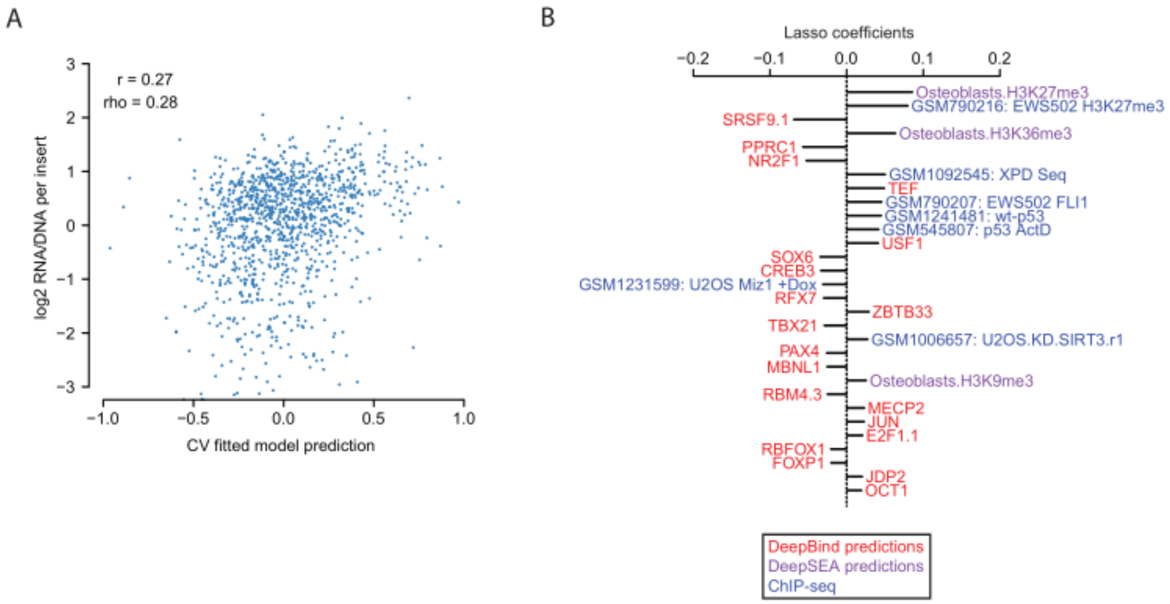

Supplementary Figure 2. A regression model to predict enhancer efficacy. A) Scatter plot showing the relationship between 10-fold cross-validated (CV) predictions derived from a lasso regression model and measured enhancer activity for 1,150 wild-type enhancers. Shown also are the Pearson (r) and Spearman (rho) correlations between the data. **B)** The top 30 coefficients derived from a lasso regression model trained on the full dataset, with ChIP-Atlas IDs replaced with sample of origin for ease of interpretation. Each feature is colored according to the origin of the dataset from which it was derived. Positive and negative coefficients lead to an increase and decrease in the predicted enhancer efficacy, respectively. Source data are provided as a Source Data file.

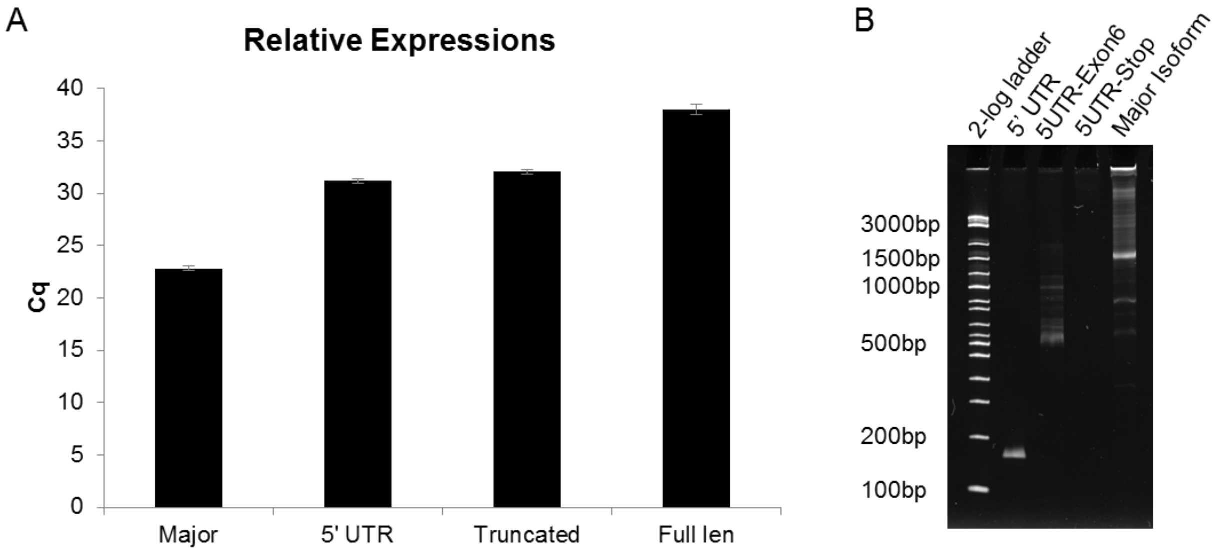

Supplementary Figure 3. Expression of different HBP1 isoforms. A) Y axis is the Cq value from RT-qPCR from SW1353 cells. The major allele uses a forward primer at the start codon and reverse primer at the conserved stop codon of the major isoforms. The 5' UTR primer set amplifies a short product, entirely within the alternative 5' UTR. The truncated primer set amplifies both ENST00000497535 and ESNT00000485846. The full length primer set includes a forward primer in the alternative TSS and reverse primer at the stop codon of the major isoform. We do not identify any full length product utilizing the alternative TSS. Error bars are one standard error. B) Gel of qPCR products. First lane is a 2-log ladder. Second lane is the 5' UTR amplification. Expected size is 156 bp. Third lane is the truncated amplification. The primer set should amplify both ENST00000497535 (expected size 548bp) and ENST00000485846 (expected size 846bp). However, ENST00000485846 contains an internal exon while ESNT00000497535 does not. We ran the PCR product on a gel and Sanger sequenced the purified PCR product, which did not include the internal exon. Fourth lane is amplifying from the alternative 5' UTR to canonical stop. There was no amplification product. Fifth lane is the major isoform (amplifying from canonical start to canonical stop. Expected size is 1,455 bp. Source data are provided as a Source Data file.

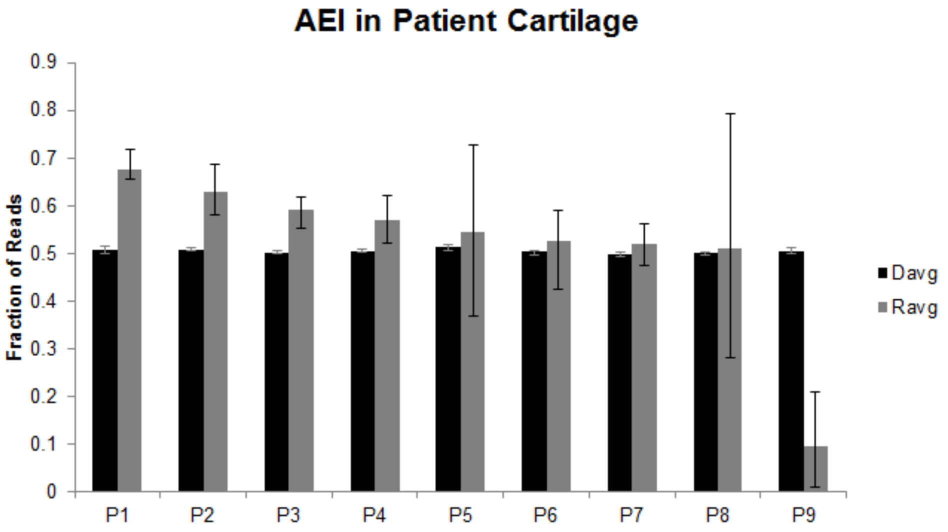

Supplementary Figure 4. Allelic Expression Imbalance (AEI) in cartilage from patients receiving total knee replacements. Black bars are the fraction of DNA reads aligning to the minor allele. Grey bars are the fraction of RNA reads aligning to the minor allele. Bars indicate the minimum and maximum fraction from three technical replicates. Source data are provided as a Source Data file.

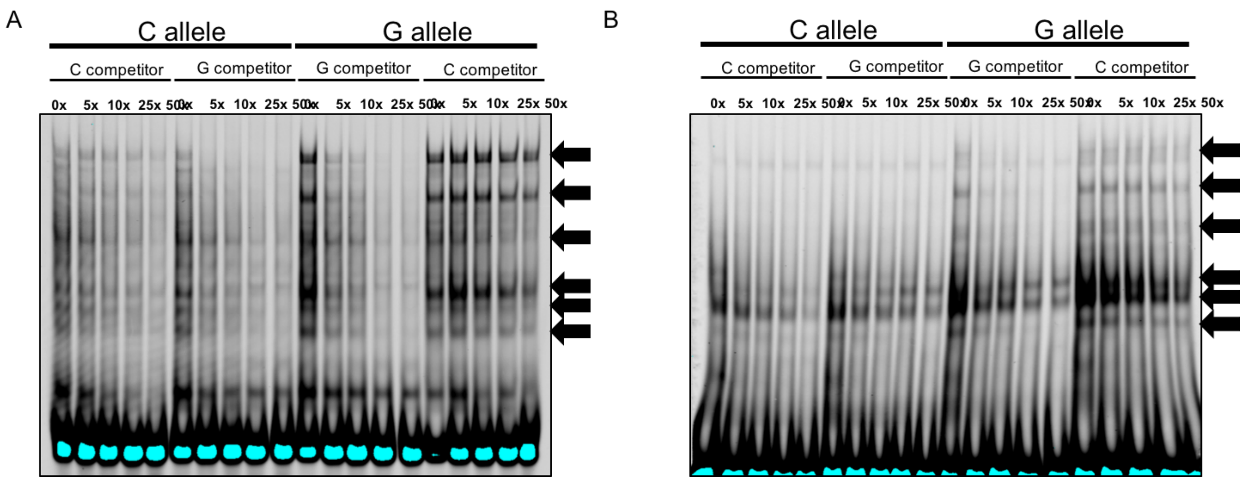

Supplementary Figure 5. Electrophoretic mobility shift assay (EMSA) analyses of expression of the rs4730222 C and G alleles. Nuclear protein extract from A) Saos-2 cells and B) SW1353 cells were used to analyze protein complex binding to rs4730222 alleles. Increasing concentrations of unlabeled C allele and the G allele competitor were added to the EMSA reactions containing cell nuclear extract and a C or G allele probe. Arrows indicate several complexes binding with much greater affinity to the G allele than to the C allele. Source data are provided as a Source Data file.

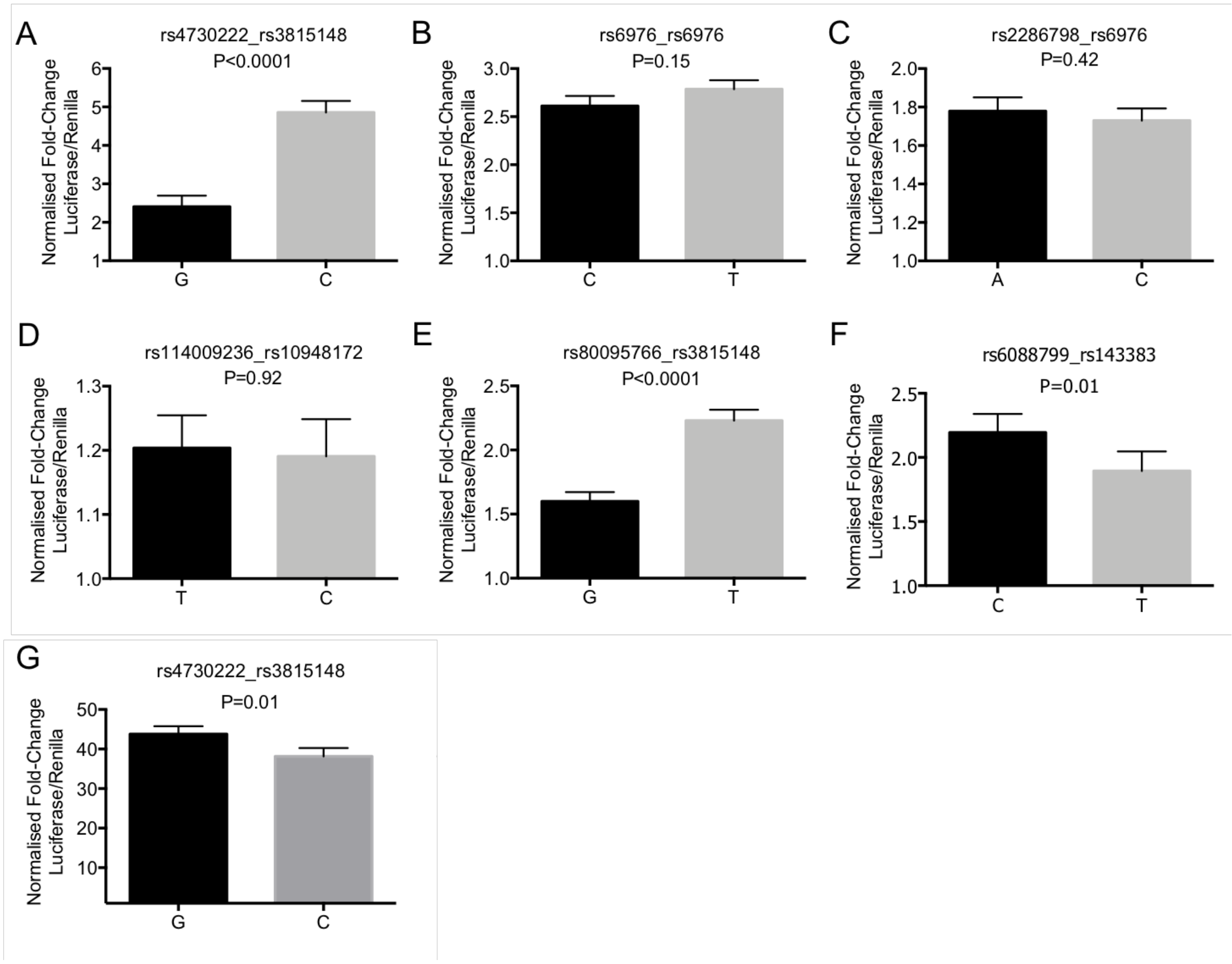

Supplementary Figure 6. Luciferase assays for six SNPs with differential regulatory activity in MPRA. A-F) rs4730222, rs6976, rs2286798, rs114009236, rs80095766, and rs6088799 cloned into the pGL3-Promoter luciferase reporter plasmid and tested as enhancers. G) rs4730222 cloned into the pGL3-Basic luciferase reporter plasmid and tested as a promoter. N=5-6 technical replicates. Source data are provided as a Source Data file.

Supplementary Table 1

List of 20 genome-wide significant (GWS) and 15 genome-wide suggestive (GWSu) variants compiled in May 2017

Lead SNP	Nearest protein coding gene	Ref	Annotation	#Linked SNPs tested	Significance
rs6976	*GLT8D1*	9	3' UTR	280	GWS
rs12107036	*TP63*	9	intronic	1	GWSu
rs10948172	*SUPT3H*	9	intronic	213	GWSu
rs9350591	*FILIP1*	9	intergenic	73	GWS
rs3815148	*HBP1*	10	intronic	284	GWS
rs4836732	*ASTN2*	9	intronic	2	GWS
rs10492367	*KLHDC5*	9	intergenic	5	GWS
rs835487	*CHST11*	9	intronic	7	GWS
rs11842874	*MCF2L*	11	intronic	8	GWS
rs225014	*DIO2*	12	exonic	19	GWSu
rs945006	*DIO3*	13	3' UTR	1	GWSu
rs3204689	*ALDH1A2*	14	3' UTR	52	GWS
rs8044769	*FTO*	9	intronic	8	GWSu
rs12982744	*DOT1L*	15	intronic	21	GWSu
rs6094710	*NCOA3*	16	intergenic	11	GWS
rs143383	*GDF5*	17	5' UTR	98	GWS
rs4764133	*MGP*	18	intergenic	137	GWS
rs3850251	*ENPP3*	18	intronic	6	GWSu
rs754106	*LRCH1*	19	intronic	10	GWSu
rs6766414	*STT3B*	19	intergenic	29	GWSu
rs2862851	*TGFA*	20	intronic	25	GWS
rs10471753	*PIK3R1*	20	intergenic	29	GWS
rs2236995	*SLBP*	20	intronic	4	GWS
rs496547	*TREH*	20	intergenic	3	GWS
rs4867568	*LSP1P3*	21	intergenic	6	GWSu
rs788748	*IGFBP3*	22	intergenic	10	GWSu
rs12901499	*SMAD3*	23	intronic	28	GWSu
rs4907986	*COL11A1*	24	intronic	23	GWSu
rs1241164	*COL11A1*	24	intronic	29	GWSu
rs833058	*VEGF*	24	intergenic	1	GWSu
rs10116772	*GLIS3*	25	intronic	8	GWS
rs2820436	*ZC3H11B*	26	intronic	43	GWS
rs11335718	*ANXA3*	26	intronic	1	GWS
rs11780978	*PLEC*	26	intronic	110	GWS
rs2521349	*MAP2K6*	26	intronic	20	GWS

Supplementary Table 2

Features selected by lasso regression (coefficients are ordered by absolute value)

Feature	Coefficient
DS.Osteoblasts.H3K27me3	0.085880834
C.SRX096367	0.079981736
DB.SRSF9.1	-0.069125859
DS.Osteoblasts.H3K36me3	0.063888124
DB.PPRC1	-0.057399308
DB.NR2F1	-0.052929165
C.SRX246954	0.050471075
DB.TEF	0.049414427
C.SRX096358	0.045939225
C.SRX361198	0.045264818
C.SRX020764	0.042120155
DB.USF1	0.041525555
DB.SOX6	-0.034834583
DB.CREB3	-0.033850879
C.SRX351402	-0.031337997
DB.RFX7	-0.030111084
DB.ZBTB33	0.029407232
DB.TBX21	-0.02927592
C.SRX188792	0.027675678
DB.PAX4	-0.0262769
DB.MBNL1	-0.025701243
DS.Osteoblasts.H3K9me3	0.025693749
DB.RBM4.3	-0.025378771
DB.MECP2	0.023313748
DB.JUN	0.023051597
DB.E2F1.1	0.021092706
DB.RBFOX1	-0.020840489
DB.FOXP1	-0.020521239
DB.JDP2	0.020518713
DB.OCT1	0.019713559
C.SRX718131	-0.018691036
DB.SP1	0.018421412
DB.SP8	0.018165204
C.SRX1304817	0.018113229
DB.RCOR1	0.017980009
DB.CNOT4	0.017740944
C.SRX369064	0.017693154
DB.IRF2	-0.017629623
DB.IRX2	-0.016892127
DB.OLIG3	0.016333897

DB.NKX2.3	-0.015182671
DB.GATA5	0.01509492
DS.Osteoblasts.H3K4me1	0.01450866
C.SRX873867	0.014115214
DB.FOXK1	0.013590787
DB.EBF1.1	-0.013269619
DB.MYBL2.1	0.012975591
DB.TBX2	-0.011331238
DB.ZNF232	-0.011276329
DB.TP53	0.011197381
DB.MSC	0.010798274
DB.JUND	0.010477138
DB.RHOXF1	-0.009772226
DB.IRF3	-0.00941078
C.SRX718133	0.0092722
DB.PBX3	0.009174918
DB.ZFP263	-0.00900421
DB.RBM28	0.008294137
DS.Osteoblasts.H3K79me2	0.00815997
DB.FOXM1	0.008102054
DB.HNRNPCL1	-0.007442816
C.SRX1003127	-0.007154884
DB.EBF1	0.006423656
DB.TCF7L1	0.005701588
DB.POU2F2	0.005377294
DB.ZEB1	-0.005083888
DB.SPI1	0.004835023
DB.NR3C1	0.004376897
C.SRX275502	0.004069566
DB.MNX1	0.003662704
DB.TEAD1	0.003641762
DB.ESRRB	0.003639884
DS.Osteobl.CTCF	-0.002823375
DB.ZNF524	0.002468316
C.SRX246953	0.002186438
DB.SCRT2	0.00130815
(Intercept)	-1.69E-16

Supplementary Table 3

RegulomeDb findings for eQTLs at rs2286798

Method	Location	Affected Gene	Cell Type	Additional Info	Reference
eQTL	chr3:52821176..52821177	WDR51A	Lymphoblastoid	cis	20220756
eQTL	chr3:52821176..52821177	FLJ12442	Lymphoblastoid	cis	17873874
eQTL	chr3:52821176..52821177	ITIH3	Lymphoblastoid	cis	18846210
eQTL	chr3:52821176..52821177	NT5DC2	Lymphoblastoid	cis	18846210
eQTL	chr3:52821176..52821177	FLJ12442	Monocytes	cis	20502693
eQTL	chr3:52821176..52821177	ITIH4	Monocytes	cis	20502693

Supplementary Table 4

RegulomeDb results ChIP-seq binding at rs6976

Method	Location	Bound Protein	Cell Type	Reference
ChIP-seq	chr3:52728576..52729497	POLR2A	GM12878	ENCODE
ChIP-seq	chr3:52728786..52729356	POLR2A	GM12891	ENCODE
ChIP-seq	chr3:52728587..52728843	ATF1	K562	ENCODE
ChIP-seq	chr3:52728610..52728890	JUND	K562	ENCODE
ChIP-seq	chr3:52728678..52728859	POLR2A	H1-hESC	ENCODE
ChIP-seq	chr3:52728667..52728911	EGR1	K562	ENCODE
ChIP-seq	chr3:52728493..52728949	JUND	K562	ENCODE
ChIP-seq	chr3:52728586..52728902	EP300	K562	ENCODE
ChIP-seq	chr3:52728567..52728923	MAX	K562	ENCODE
ChIP-seq	chr3:52728613..52728992	POLR2A	HepG2	ENCODE
ChIP-seq	chr3:52728598..52728894	RCOR1	K562	ENCODE
ChIP-seq	chr3:52728573..52728909	TBL1XR1	K562	ENCODE

Supplementary Table 5

Primers used to create EMSA probes targeting both alleles of rs4730222. The polymorphic base is underlined in each primer

SNP	Allele	Forward primer (5'-3')	Reverse primer (5'-3')
rs4730222	C	TGAATGGCGAAAGAGCGTGGGGGATGGACTT	AAGTCCATCCCCCACGCTCTTTCGCCATTCA
	G	TGAATGGCGAAAGAGGGTGGGGGATGGACTT	AAGTCCATCCCCCACCCTCTTTCGCCATTCA

Supplementary Table 6

853 features considered in a model trained to predict enhancer activity.
Description of features considered, feature type (i.e., computationally predicted or experimentally derived), data source, and number of features in the category.

Feature description	Feature type
GC/CpG content	Sequence-derived
Mean primate, mammalian, and vertebrate conservation	Sequence-derived
Predicted histone marks, DNase hypersensitivity, and TF binding in Osteoblast cells	Sequence-derived
Predicted score of TF binding for human, mouse, chicken, frog, fugu, and zebrafish models	Sequence-derived
Mean signal of TF and histone marks in bone-related cell types	ChIP-seq

Supplementary Table 7

SNP	Forward Primer (5'-3')	Restriction Site	Reverse Primer (5'-3')	Restriction Site
rs4730222	TCT GGC TGA GCT GAG GAG	KpnI	ATT ACC TCG CTG TCA GTG CT	XhoI and HindIII
rs6976	AGA TCA GGC CAC AGG TTA CA	KpnI	AGG CTG CCA AGT TAC TCC AC	XhoI
rs228798	TCG CTG GAC TAT GGG TTT GT	KpnI	TGC CTC CTC ACA GAC ATA CA	XhoI
rs114009236	GCA AGT CAG TTC AGC CTT TCT	KpnI	GCC TTG AAC TCC TGA CCT CA	XhoI
rs80095766	TGA AAC GTC ATC ATG CCA AAA	KpnI	AGC CAA CTT TTC CTG TAC GC	XhoI
rs6088799	TGC TGC TCA CTC TTT GGG T	KpnI	TCC TTG ATC ACT GGC AAC CT	XhoI
