# Supplementary material for: Functional testing of thousands of osteoarthritis-associated variants for regulatory activity
Source: Nat Commun. 2019 Jun 4;10:2434. doi: 10.1038/s41467-019-10439-y (PMC6547687; doi:10.1038/s41467-019-10439-y)
Supplement: Supplementary file 2 — Description of Additional Supplementary Files [file 41467_2019_10439_MOESM2_ESM.docx]

**Description of Additional Supplementary Files**

Supplementary Data 1. Array synthesized oligos for each allele. Contains SNP, GRCh37 position, allele, lead SNP, and synthesized sequence.

Supplementary Data 2. Activity scores for all SNPs with at least three independent measurements.

Supplementary Data 3. Scores and Mann Whitney uncorrected p-value for 1132 SNPs.

Supplementary Data 4. Scores for each allele from three independent transfections.

Supplementary Data 5. 853 evolutionary, biochemical, and sequence derived features used to train the lasso regression model.

Supplementary Data 6. Lasso regression model on the entire dataset.

Supplementary Data 7. RegulomeDb results for ChIP-seq binding at rs4730222.
